# Production scheduling of prefabricated components considering delivery methods

**DOI:** 10.1038/s41598-023-42374-w

**Published:** 2023-09-12

**Authors:** Shuqiang Wang, Xi Zhang

**Affiliations:** 1 School of Civil Engineering, Architecture and Environment, Hubei University of Technology, Wuhan, 430068 People’s Republic of China; 2 Innovation Demonstration Base of Ecological Environment Geotechnical and Ecological Restoration of Rivers and Lakes, Hubei University of Technology, Wuhan, 430068 People’s Republic of China

**Keywords:** Civil engineering, Computer science

## Abstract

To address the processing scheduling problem involving multiple molds, components, and floors, we propose the Genetic Grey Wolf Optimizer (GGA) as a means to optimize the production scheduling of components in a production line. This approach combines the Grey Wolf algorithm with the genetic algorithm. Previous methods have overlooked the storage requirements arising from the delivery characteristics of prefabricated components, often resulting in unnecessary storage costs. Intelligent algorithms have been demonstrated to be effective in production scheduling, and thus, to enhance the efficiency of prefabricated component production scheduling, our study presents a model incorporating a production objective function. This model takes into account production resources and delivery characteristics constraints. Subsequently, we develop a hybrid algorithm, combining the grey wolf algorithm with the genetic algorithm, to search for the optimal solution with a minimal storage cost. We validate the model using a case study, and the experimental results demonstrate that GAGWO successfully identifies the best precast production schedule. Furthermore, the precast production plan, considering the delivery method, is found to be reasonable.

## Introduction

The 14th Five-Year Plan highlights the goal of achieving a proportion of over 30% for assembled buildings in new construction. In 2020, the national new construction of assembled buildings amounted to 629 million square meters, while in 2021, it reached 740 million square meters, representing a growth rate of 17.6%. Considering the growth rate data and planning requirements, it is projected that by 2025, the new construction of assembled buildings will reach an estimated area of 1.2 billion m^2^. The production of assembled building components plays a crucial role in the development of assembled buildings, making the development of production scheduling for these components economically and practically significant. As China actively promotes the advancement of assembled buildings, the scheduling of prefabricated component production has gained increasing attention^1^.

Existing studies on production scheduling algorithms in precast component production consider various practical constraints such as manpower, tooling solutions, and materials to determine the most efficient solution in terms of duration or cost. However, in actual construction projects, it is essential to closely coordinate the production and construction schedules of prefabricated components, particularly when coordinating prefabricated components of the same standard layer to ensure their delivery as a unified task. Unfortunately, the current research lacks a comprehensive examination of the optimal solution that incorporates the unique characteristics of the production delivery method of assembled buildings, specifically whole floor delivery. The time required for prefabricated component production may sometimes cause delays in meeting the construction schedule, leading to reputation and efficiency losses for enterprises. Conversely, if prefabricated components are completed too early and require on-site stacking, it increases the inventory cost for production, which should not be overlooked in cost calculations. Regrettably, there are few studies that specifically address the production storage cost based on the delivery characteristics of production projects. To tackle these challenges, this study combines the constraints imposed by whole layer delivery characteristics and employs the GAGWO algorithm for production scheduling optimization, aiming to select the solution with the lowest storage cost. Considering the rapid development of utilizing intelligent algorithms to solve production scheduling problems for prefabricated components, this paper proposes an enhancement of the Grey Wolf Algorithm (GWO) by integrating it with the Genetic Algorithm (GA). GWO is a well-known algorithm, while GA is a relatively new algorithm, and both possess significant advantages in solving production scheduling problems. Utilizing appropriate formulations, metaheuristic algorithms offer effective solutions for production scheduling problems, particularly the Grey Wolf algorithm, which demonstrates high accuracy and fast convergence. However, similar to other intelligent optimization algorithms, it faces the challenge of decreasing population diversity over time, resulting in local optimization. Therefore, this study proposes the integration of a GA into the grey wolf algorithm to update the population and introduce genetic operators to generate more diverse offspring, thereby avoiding convergence to local optima. Previous researchers have made modifications to the grey wolf algorithm for various production scheduling objectives, often combining it with the Simulated Annealing algorithm to achieve favorable outcomes. Given the usefulness of heuristic algorithms in production scheduling, this paper aims to demonstrate the superiority of the Genetic Grey Wolf algorithm for the research problem at hand. To achieve this, the optimized production scheduling results obtained using the genetic grey wolf algorithm are compared with those obtained using other algorithms such as simulated annealing, genetic algorithm and grey wolf algorithm. Through this comparison, the study aims to provide evidence and explore the relationship between lead time, inventory cost, and the optimization algorithms employed.

The remaining sections of this paper are structured as follows. In “Selection of constraints for scheduling production of precast concrete components” section, we conduct a comprehensive review of related research. In “Production scheduling model for precast concrete components” section encompasses the problem description, formulation, and model development. We proceed to introduce the grey wolf algorithm combined with the genetic algorithm for tackling the production scheduling problem in “Case study” section. In “Conclusion” section presents a series of numerical analyses utilizing real production cases. It compares the results of the genetic grey wolf algorithm with those obtained using other intelligent algorithms to highlight the superior performance of the genetic grey wolf algorithm in addressing production scheduling problems that incorporate delivery characteristics. Lastly, Sect. 6 provides concluding remarks summarizing the contributions of this study, as well as discussing its limitations and future prospects.

## Selection of constraints for scheduling production of precast concrete components

Various scholars have provided their perspectives on considering production constraints. Chan and Hu^2,3^ examined the production methods and processes of precast components, categorizing them based on whether the production process is interruptible. Leu and Hwang^4^ argued that precast component production in plants can resemble repetitive production in traditional shops, emphasizing the inclusion of mechanical and manual constraints to improve resource utilization. Ko^5,6^ further considered the buffer zone size between different processes of precast component production and developed a multi-objective scheduling model (MOPSPSM) that accounted for buffers between machines and various production resources. However, these studies did not address material inventory and tooling quantities that are constrained by the production of prefabrication plants, as well as cost elements during field installation.

Wang Chaojing et al.^7^ identified the incomplete nature of Chan and Hu’s model and introduced storage and transportation processes to minimize delay and early delivery costs. Wang^8^ approached the production scheduling problem from a supply chain perspective, considering mold manufacturing, PC storage, and transportation constraints on prefabricated component production. Lu^9^ proposes a Pareto-based collaborative multi-objective optimization algorithm with the objective of minimizing the maximum completion time and total energy consumption, and develops three operators to guide the collaborative search of the algorithm based on the problem attributes. Anvari^10^ constructed a flexible job shop model for prefabricated components, optimizing the entire production–transportation-assembly process using a multi-objective genetic algorithm to minimize completion time and production costs. These studies incorporated storage and transportation constraints to enhance resource allocation. However, the inventory cost impact based on the delivery method perspective was not considered. The order constraint is to produce and deliver a set of products as a whole. Geng^11^ uses the Memetic algorithm to solve for the maximum completion time and total energy cost, and designs three heuristic rules and neighborhood search strategies based on workpiece order and completion time. Xie^12^ treated precast components within the same standard layer as a single order, optimizing two-stage delivery to align with the construction schedule. However, this approach had limitations and did not account for the lifting sequence of standard layers, potentially leading to delayed assembly on-site. Building upon previous studies, this research considers mold, labor, and whole floor delivery constraints for production scheduling optimization, serving as a practical guide for actual production requirements.

### Application of grey wolf algorithm in production scheduling

The grey wolf optimization (GWO) algorithm, proposed by Seyedali et al.^13^, is a population-based intelligence optimization method that models the foraging behavior and hierarchical structure of grey wolf populations. Compared to particle swarm and genetic algorithms, GWO exhibits fast convergence, high accuracy, and ease of implementation due to its small control parameters. However, it is prone to falling into local optima. To address this limitation, several scholars have conducted relevant research. Lu^14^ introduced a multi-objective cellular GWO algorithm to solve the hybrid flow shop scheduling problem considering noise pollution. By incorporating meta-cellular automaton and variable neighborhood search, the algorithm expanded the search space and improved local search capability. Wu^15^ proposed a new mechanism for generating grey wolf populations to address flexible shop floor scheduling problems, enhancing population diversity. They also introduced a sentinel wolf method, combining genetic and simulated annealing algorithms to accelerate convergence. Luo^16^ developed a binary GWO algorithm for the multidimensional knapsack problem, incorporating the differential evolution operator for individual updates and an optimal solution retention mechanism. This mechanism only replaces the population when a new solution surpasses the current optimal solution. Gölcük^17^ proposed a binary GWO algorithm, employing multi-parent hybridization in the genetic algorithm for binary problems. They introduced a discrete adaptive mutation mechanism to increase population diversity, validated on set-and-backpack problems. Lu^18^ designed an improved social hierarchy for GWO, embedding genetic operators to enhance the algorithm’s search capability. They also incorporated a transformation law into the update operator for neighborhood transformation and developed a hybrid multi-objective GWO algorithm for scheduling optimization. In summary, the GWO algorithm offers advantages such as a simple search mechanism, fast convergence, and strong global search capability. In this paper, we apply the hybrid genetic grey wolf algorithm to address the integrated optimization problem of production scheduling.

## Production scheduling model for precast concrete components

**Statement:** The individual surveys and precast plant production data involved in this paper have received approval from the participants and the CCBC Hannan Precast Plant to support the study.

The optimization problem of scheduling prefabricated building components represents a novel form of production scheduling challenge that builds upon the classical FSSP. This problem incorporates resource constraints and production delivery characteristics, making it more closely related to the traditional flow shop problem rather than the conventional building production problem. Addressing this issue necessitates the development of a mathematical model and solution that effectively account for the unique characteristics of production delivery and resource constraints within the process.

### Flow process of prefabricated component production

Warszawski^19^ attempted to address the precast production scheduling problem using a flow shop production scheduling model. The conventional precast production scheduling model is based on a mobile assembly line production system, which represents the direct production process of precast components and aims to optimize their production process.

**Figure 1:**
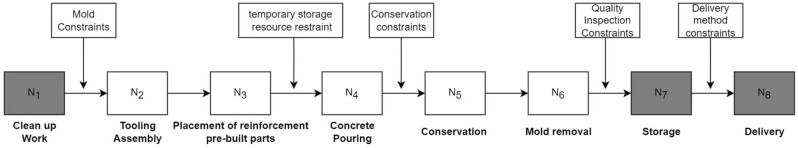
Production process of prefabricated components.

The conventional precast production scheduling model focuses solely on precast production preparation and post-production storage, overlooking the actual production and delivery process. This limitation may result in longer production cycles and increased storage costs. Consequently, the traditional production scheduling model does not align with actual production requirements and fails to provide appropriate guidance. To address this issue, this paper introduces an expanded production process consisting of eight stages, based on the traditional production scheduling model: mold handling, steel precast installation, concrete placement, concrete maintenance, mold removal, finished product quality inspection, storage, and delivery, as illustrated in Fig. 1.

### Production scheduling model for precast concrete components

**Figure 2:**
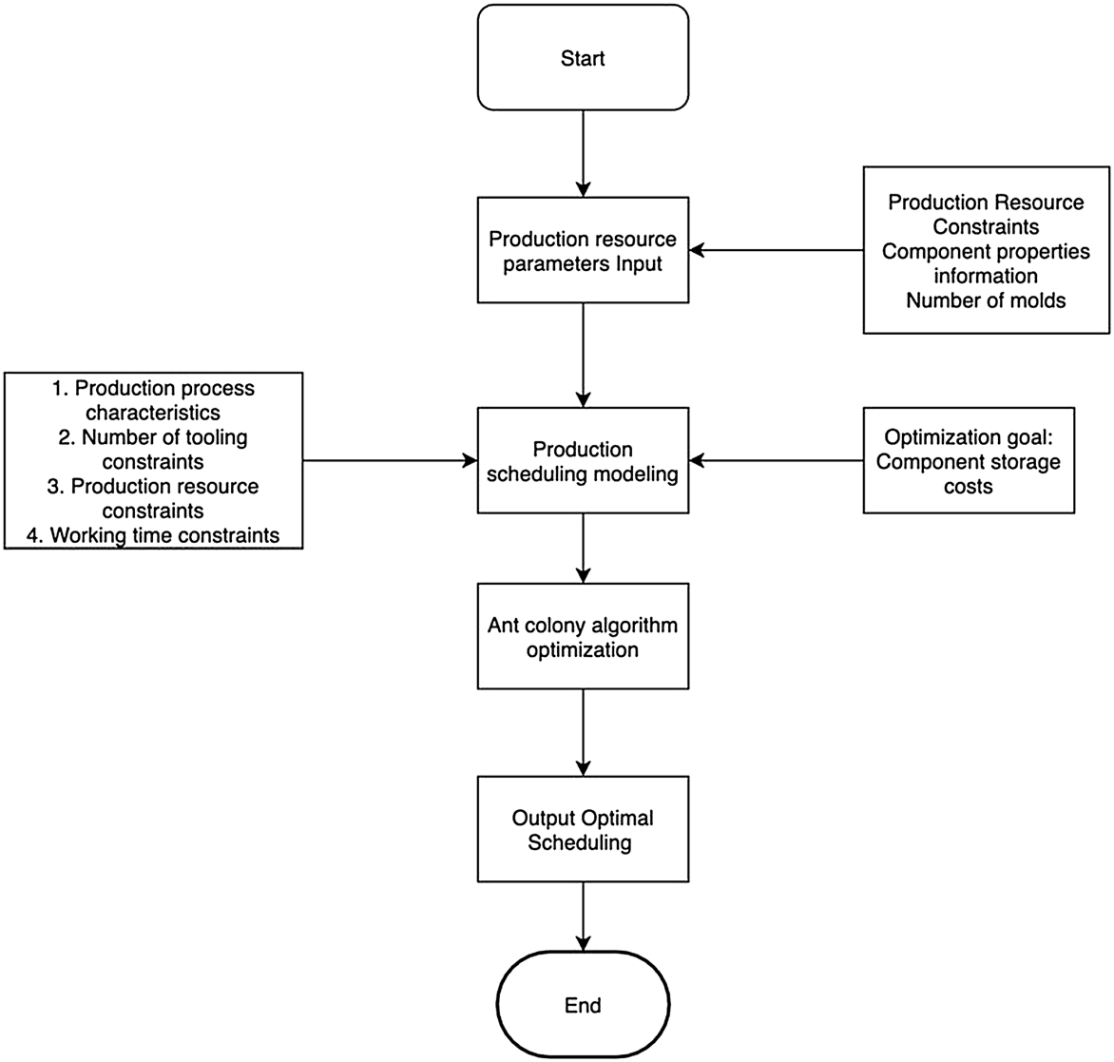
Precast concrete component production scheduling model.

Drawing on the traditional production scheduling model, the scheduling model for precast concrete component production is developed as a replacement flow shop model, which incorporates process characteristics and resource constraints. Building upon the aforementioned description and assumptions, the mathematical model for precast component production scheduling is formulated and presented in Fig. 2.

#### Prefabricated component production delivery characteristics constraints

The conventional production scheduling model assumes the delivery of prefabricated components on a piece-by-piece basis. However, in practice, prefabricated components are often delivered in batches, known as whole floor delivery. The production of components using whole floor delivery entails varying delivery times based on the specific buildings they are intended for. These variations in delivery times have implications for inventory costs. Consequently, it is necessary to ensure that the delivery time for each component is met.

1 $${E}_{Tij}\le {E}_{Ti(i-1)}$$

where ${E}_{Tij}$ is the delivery time of the jth component of the ith building, and ${S}_{Tij}$ is the time when the production of the jth component of the ith building is completed. m is the number of building components and n is the number of buildings. The constraint Eq. (1) is formulated to enforce that each prefabricated component can only be delivered for a specific floor once the prefabricated components of the preceding floor have been delivered.

**Figure 3:**
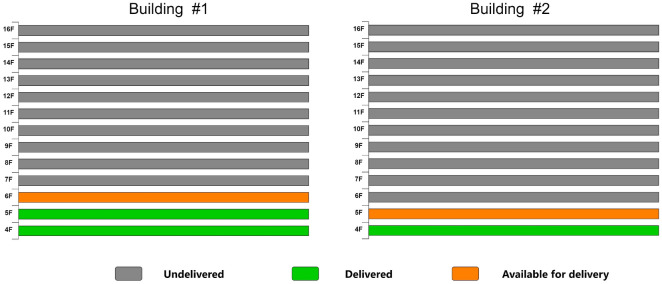
Completion of production of target layer components.

In the actual production process, the completion of producing components for a specific layer is closely tied to the project schedule. To prevent the prefabricated components from occupying the construction site, the delivery and lifting of components from the preceding floors must be completed before the components from the subsequent floors can be delivered and lifted. For example, once all the components for the fifth floor of Building 1 and the fourth floor of Building 2 are delivered, the components for the sixth floor of Building 1 and the fifth floor of Building 2 become eligible for delivery (Fig. 3).

**Figure 4:**
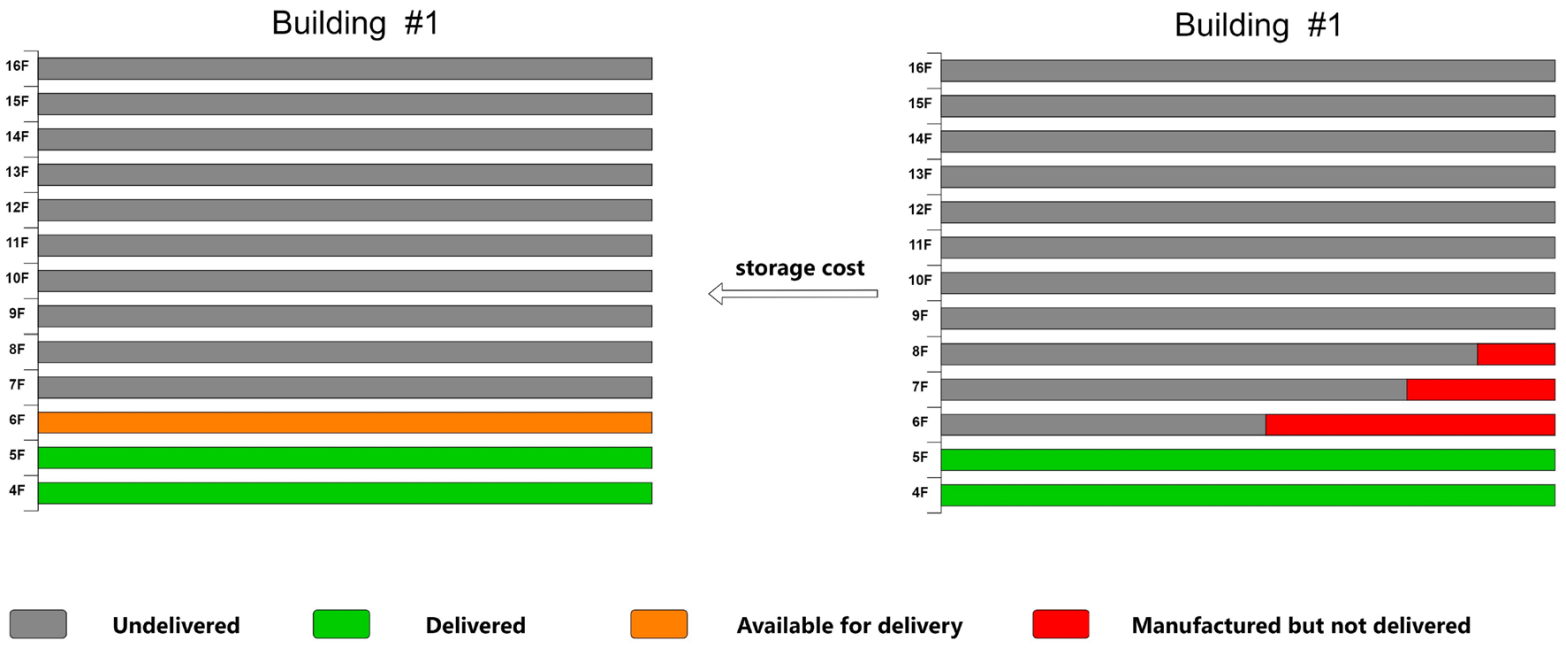
Unfinished production of target layer components.

The delivery of prefabricated components is time-sensitive, and to mitigate transportation costs, the factory employs a whole-floor delivery approach in the actual production and delivery process. In the provided figure, although the sixth floor of Building 1 has been delivered, the production of prefabricated components for the target floor is not yet complete, resulting in a delay in their delivery. Consequently, these components are stored in the factory warehouse along with the components for the seventh and eighth floors, which have been produced but not yet delivered, incurring storage costs (Fig. 4).

#### Labor resource constraints

In the production and processing of prefabricated components, the efficiency of production workers plays a crucial role in determining the completion time of these components. To adhere to the regulations outlined in the Chinese labor law, this study categorizes workers’ time into working hours and non-working hours. Within the limited time frame, workers are permitted to arrange overtime work as needed. Any tasks that remain unfinished during normal working hours can be completed during overtime. However, tasks that exceed the allotted overtime hours will need to be continued on the following day.

2 $$ WT_{j,i} = \left\{ {\begin{array}{*{20}l} {C\left( {J_{{j - B_{i} }} ,N_{i + 1} } \right) - P_{{j - B_{i} ,i + 1}} - C\left( {J_{j} ,{\text{N}}_{i} } \right),} \hfill \\ {0,} \hfill \\ \end{array} } \right. $$

where ${WT}_{j,i}$ represents the waiting time for jth component to be ready to be sent to the buffer in the ith process. ${B}_{i}$ is the buffer space between the ith work station and the i + 1th work station. In case, when the completion time of the jth component in the ith process is greater than or equal to the start time of the jth − ${B}_{i}$. When the start time of the jth component in the i + 1st process is greater than or equal to the start time of the jth component in the i + 1st process, the buffer space is ${B}_{i}$. If the buffer space is not fully occupied; otherwise, there will be a waiting time due to insufficient buffer space ${B}_{i}.$ This is determined by the layout of the space between stations in the assembly line. $\mathrm{C}({\mathrm{J}}_{\mathrm{j}},{\mathrm{N}}_{\mathrm{i}})$ is the processing completion time of jth component in the ith process. ${P}_{j,i}$ is c is the processing time of the jth component in the i-th process.

#### Mold resource constraints

Mold resources play a crucial role in the production of precast components, but their availability is limited due to factors such as high production costs, low versatility, and limited capacity. Consequently, in the production process, prefabricated components often have to wait until the previous batch of components is demolded to free up the molds, resulting in mold waiting time.

**Figure 5:**
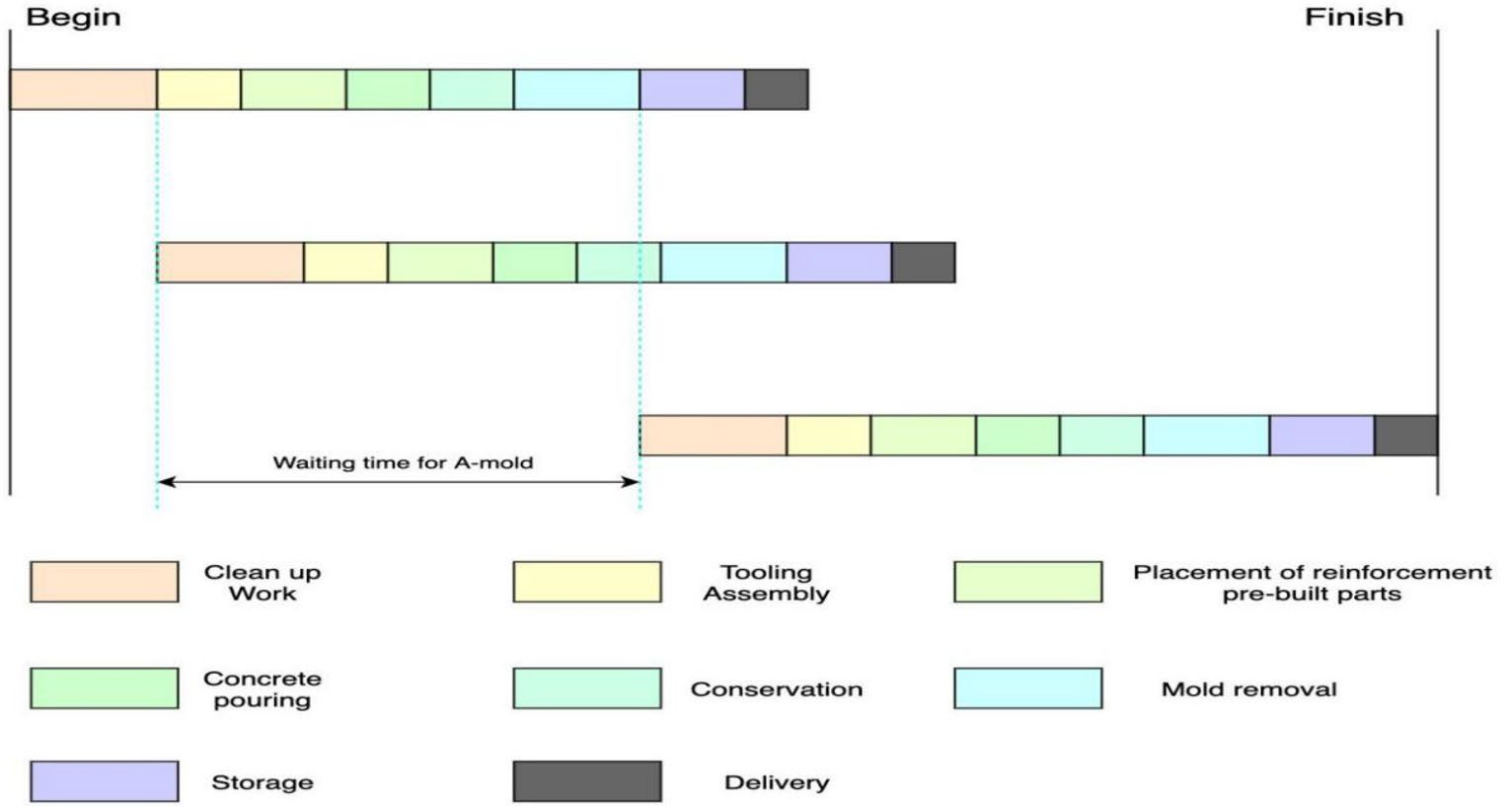
Mould constraints for prefabricated component production.

When arranging the production of precast components, the limited number of molds poses a challenge. For instance, in Fig. 5, both precast member No. 1 and precast member No. 2 require a-type molds. However, due to the limited availability of a-type molds, production of member No. 2 can only commence after the demolding of member No. 1 is completed and the a-type molds become available. As a result, the a-mold waiting time for component j occurs.

3 $$C({J}_{j,a},{N}_{0})=min{X}_{a}\{\forall y\{C({J}_{y,a},{N}_{5})\}\}$$

where ${\mathrm{X}}_{\mathrm{a}}$ is the number of a-type molds.

### Analysis of the work sequence of prefabricated parts production

#### Interruptible processes

**Figure 6:**
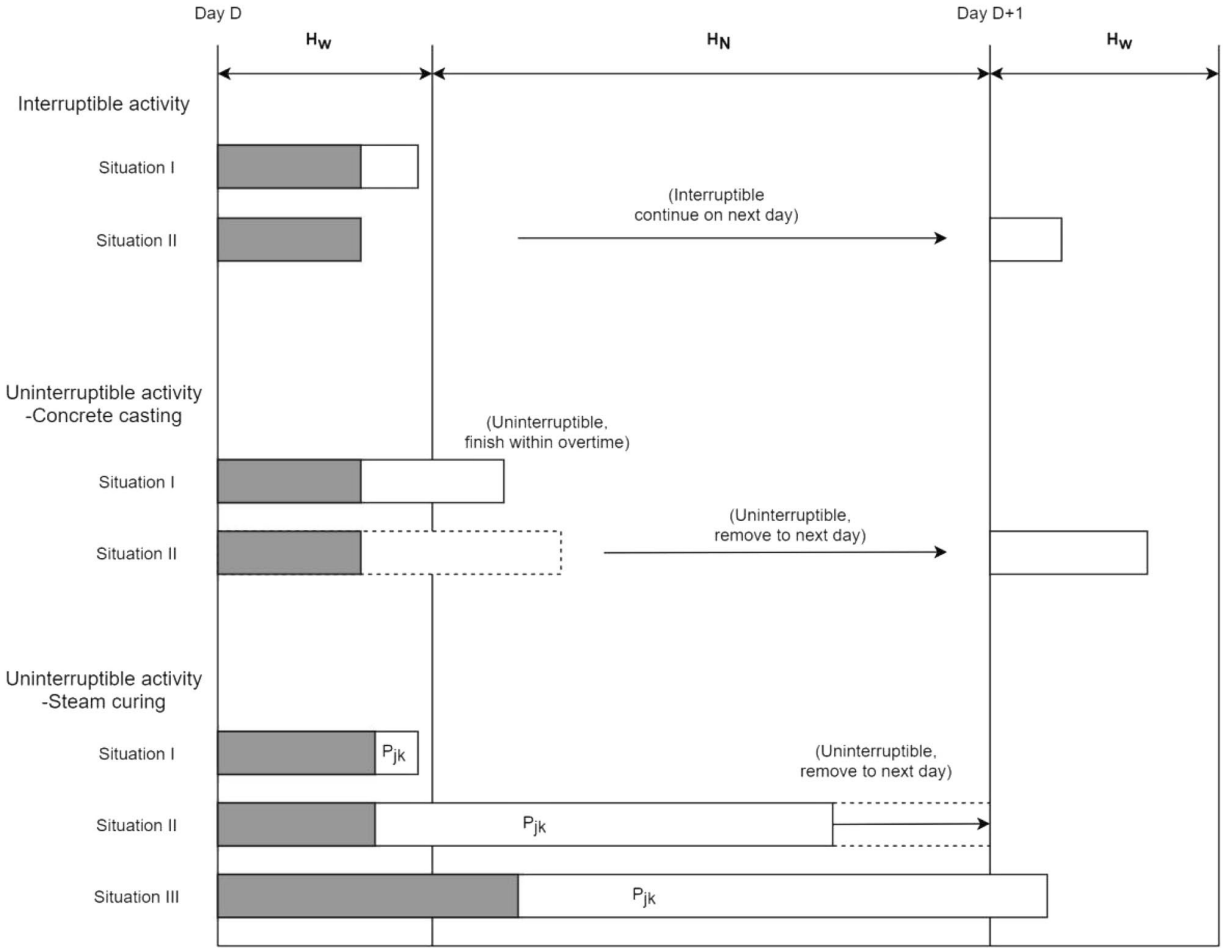
Production process characteristics.

Based on the characteristics of prefabricated component production processes, they can be broadly classified into two categories: interruptible processes and non-interruptible processes. As depicted in Fig. 1, the processes of cleaning work, mold assembly, placement of reinforcement pre-installation, and mold removal are all examples of interruptible processes. The completion time of precast component j in the ith process (station) is

4 $$ C\left( {J_{j} ,N_{i} } \right) = \left\{ {\begin{array}{*{20}l} {T,T \le 24D + H_{w} } \hfill \\ {T + H_{M} ,T > 24D + H_{W} } \hfill \\ \end{array} } \right. $$

where i = 1, 2, 3, 6; ${H}_{W}$ is the daily normal working time; ${\mathrm{H}}_{\mathrm{M}}$ is the daily non-normal working time; D is the working day, D = Integer (T/24), 24D is the total number of working hours of the whole day except the last day, T is the accumulated completion time, as shown in Fig. 6.

5 $${T}_{Z}=M\mathit{ax}\{C({J}_{j-1},{N}_{i}),C({J}_{j},{N}_{i-1})\}+{P}_{j,i}$$

#### Non-interruptible processes

1. Concrete casting process

In the case where the task can be accomplished within the permitted overtime duration, the individual is required to work overtime to complete the pouring process before leaving. However, if the pouring process cannot be completed within this timeframe, it must be deferred until the following working day. The completion time of the pouring process is as described above.

6 $$ C\left( {J_{j} ,N_{i} } \right) = \left\{ {\begin{array}{*{20}l} {T,T \le 24D + H_{w} + H_{AL} ;} \hfill \\ {24\left( {D + 1} \right) + P_{j,i} ,T > 24D + H_{W} + H_{AL} ;} \hfill \\ \end{array} } \right. $$

where i = 4. ${\mathrm{H}}_{\mathrm{AL}}$ is the allowed overtime and ${H}_{AL}<{H}_{\mathrm{M}}.$

(2) Maintenance process

In contrast to concrete pouring, the maintenance of prefabricated components is a non-interruptible process that does not typically require additional labor resources. Moreover, if the maintenance kiln has sufficient space, multiple components from the same batch can be simultaneously maintained. The start time of the maintenance process is dependent solely on the completion time of the preceding process, which in this case is pouring.

7 $${T}^{*}=C({J}_{j},{N}_{3})+{P}_{j,4}$$

${T}^{*}$ is c the start time of the maintenance process. As illustrated above, the maintenance process does not consume overtime hours even when conducted during off-hours. Therefore, the completion time of the maintenance process is defined as follows:

8 $$ C\left( {J_{j} , N_{i} } \right) = \left\{ {\begin{array}{*{20}l} {24\left( {D + 1} \right),\left( {24D + H_{W} } \right) < T^{*} < 24\left( {D + 1} \right);} \hfill \\ {T^{*} , T^{*} \le 24D + H_{w} orT^{*} \ge 24\left( {D + 1} \right)} \hfill \\ \end{array} } \right. $$

where i = 5.

#### Temporary storage resource restraint

The primary function of the temporary storage is to support continuous production on the production line. However, due to the nature of prefabricated component production, it is not feasible to have a traditional library set up near each workshop. The production line operates in real-time without any interruptions. Nevertheless, material take-off during actual production often deviates and requires accounting and timely replenishment to prevent delays. The specific calculations for this process are as follows:

9 $$ C\left( {J_{j} , N_{4} } \right) = \left\{ {\begin{array}{*{20}l} {\max \{ C\left( {J_{j - 1} , N_{3} } \right),C\left( {J_{j} , N_{3} } \right)\} + P_{j,4} , } \hfill & {D_{j} < D_{T} ;} \hfill \\ {C\left( {J_{j - 1} , N_{3} } \right) + E_{j,3} + P_{j,4} } \hfill & {D_{j} > D_{T} ;} \hfill \\ \end{array} } \right. $$

where ${E}_{j,3}$ is the Preparation time of the temporary storage resource restraint required for the member, the ${D}_{T}$ is the remaining temporary storage resource. The wire-side library experiences a resource constraint primarily concerning reinforcement cage tying and concrete preparation. Assembling the reinforcement cage with the mold is necessary, and concrete preparation should not be prolonged. In cases where these resources are insufficient, additional preparation is required, resulting in extended completion time as follows.

#### Quality inspection restraint

To maintain the quality of prefabricated components during production, a random inspection is conducted after the demoulding process. The components must meet the required quality standards before production activities can proceed. This ensures the stable and efficient operation of the production line. The calculation formula for this quality inspection is as follows:

10 $$ S\left( {J_{j} ,N_{7} } \right) = \left\{ {\begin{array}{*{20}l} {\max \{ C\left( {J_{j - 1} , N_{6} } \right),C\left( {J_{j} ,N_{6} } \right)\} + P_{j,6} + E_{j,6} } \hfill & {j\dot{ \in }{\text{P}};} \hfill \\ {\max \{ C\left( {J_{j - 1} , N_{6} } \right),C\left( {J_{j} ,N_{6} } \right) \} + P_{j,6} } \hfill & {j\dot{ \in }{\text{Q;}}} \hfill \\ \end{array} } \right. $$

where $S({J}_{j},{N}_{7})$ refers to the Storage start time for Storaging for member j. ${E}_{j,6}$ represents the time required for quality inspection, $\mathrm{P}$ represents the set of components to be inspected, and $\mathrm{Q}$ represents the set of components not to be inspected.

#### Objective function of prefabricated component production scheduling

In precast production scheduling, there are multiple optimization objectives, with cost minimization and completion time reduction being the primary concerns for all stakeholders. Therefore, in this study, the selected optimization objective is to minimize the time difference between component production completion and component production delivery, ultimately aiming to minimize the storage cost associated with precast production.

11 $$ {\text{Min}}\;\mathop \sum \limits_{{{\text{i }} = { }0}}^{{\text{n}}} \left( {\mathop \sum \limits_{{{\text{j }} = { }0}}^{{\text{m}}} \left( {{\text{E}}_{{{\text{Tij}}}} - {\text{S}}_{{{\text{Tij}}}} } \right){\text{*C}}_{{\text{i}}} } \right) $$

The conditions required for this objective function to be satisfied are

12 $${S}_{{T}_{sij}}>{E}_{Ti(j-1)}$$

where ${S}_{{T}_{sij}}$ represents the start time of the jth process of the ith component, and ${E}_{Ti(j-1)}$ represents the j-1th process end time of the i component, and Eq. (12) is designed to ensure that the immediate preceding process is completed when the current process begins.

### Genetic grey wolf algorithm to solve production scheduling model

#### Encoding and decoding

In this study, the production of five prefabricated components is used as an example. To determine the scheduling order of these components, a random key approach is employed. Random values ranging between 0 and 1 are assigned to each component, with higher values indicating higher scheduling priority (Table 1). The set of consecutive random values representing the components in the population is then arranged in descending order to establish a discrete production order.

**Table 1:** Coding method.

Gene position	${\mathrm{j}}_{1}$	${\mathrm{j}}_{2}$	${\mathrm{j}}_{3}$	${\mathrm{j}}_{4}$	${\mathrm{j}}_{5}$
Genetic content	0.41	0.32	0.56	0.34	0.85

The decoding process is divided into two stages: component sequencing and mold assignment. In the component sequencing stage, the components are arranged in the order specified by the code, considering the sequence of components before and after processing. In the mold assignment stage, the principle of first mold availability is followed. This means that when prefabricated components complete the demolding process, components that match the available molds are selected for production (Supplementary Information).

#### Initial populations

To represent Pop1, the initial population of size N is randomly generated using the 3.4.1 decoding method, which corresponds to the minimum inventory cost problem. The construction of the initial solution plays a crucial role in both the convergence speed and solution quality of the algorithm. A well-constructed initial solution can greatly enhance the algorithm’s performance. Consequently, heuristic rules are employed to generate individuals that correspond to the population Pop1.

#### Degree of adaptation

The fitness function serves as the criterion for evaluating the quality of an individual in the population. A higher value of the fitness function indicates that the individual’s solution is closer to the optimal solution and increases its likelihood of being selected. In the context of the prefabricated component flow shop scheduling problem, the objective is to minimize the minimum inventory cost (IC) associated with the production and delivery of all components. Therefore, the fitness function is defined as follows:

13 $${\mathrm{F}}_{\mathrm{i}}=\frac{1}{{\mathrm{IC}}_{\mathrm{i}}}$$

#### Grey wolf location update

“Surrounding the prey” in the grey wolf algorithm refers to the process where grey wolves gradually approach the prey and encircle it when it is released. During the optimization process of the algorithm, “hunting” involves selecting the three best grey wolves (α, β, and δ) in the current pack. The leader, α, is responsible for making decisions, while β assists the leader in decision-making. δ follows the decision orders of α and β to update the positions of other search agents based on their position information. The grey wolves randomly move within the vicinity of the prey in order to gradually approach the optimal solution. The position update formula in the grey wolf algorithm is as follows:

14 $$\mathrm{D}={\mathrm{CX}}_{\mathrm{p}}(\mathrm{t})-\mathrm{X}$$

15 $$\mathrm{X}(\mathrm{t}+1)={\mathrm{X}}_{\mathrm{p}}(\mathrm{t})-\mathrm{AD}$$

16 $$\mathrm{A}=2{\mathrm{ar}}_{1}-\mathrm{a}$$

17 $$\mathrm{C}=2{\mathrm{r}}_{2}$$

where t is the number of iterations. $\mathrm{X}(\mathrm{t})$ is the current position of the grey wolf;${\mathrm{X}}_{\mathrm{p}}$ is the position of the prey; A and C denote the coefficients of the synergy effect, respectively.${\mathrm{r}}_{1} , {\mathrm{r}}_{2}$ is a random number between [0,1]; the value of A decreases linearly with the increase of the number of iterations and finally becomes 0.

#### Individual updates

The fundamental grey wolf algorithm relies on the guidance of the current optimal individual during the individual update phase. If the optimal individual is in proximity to the global optimum, the algorithm can effectively search for the global optimal solution. However, if the optimal individual is distant from the global optimum, the algorithm may become susceptible to getting trapped in local optima.

**Figure 7:**
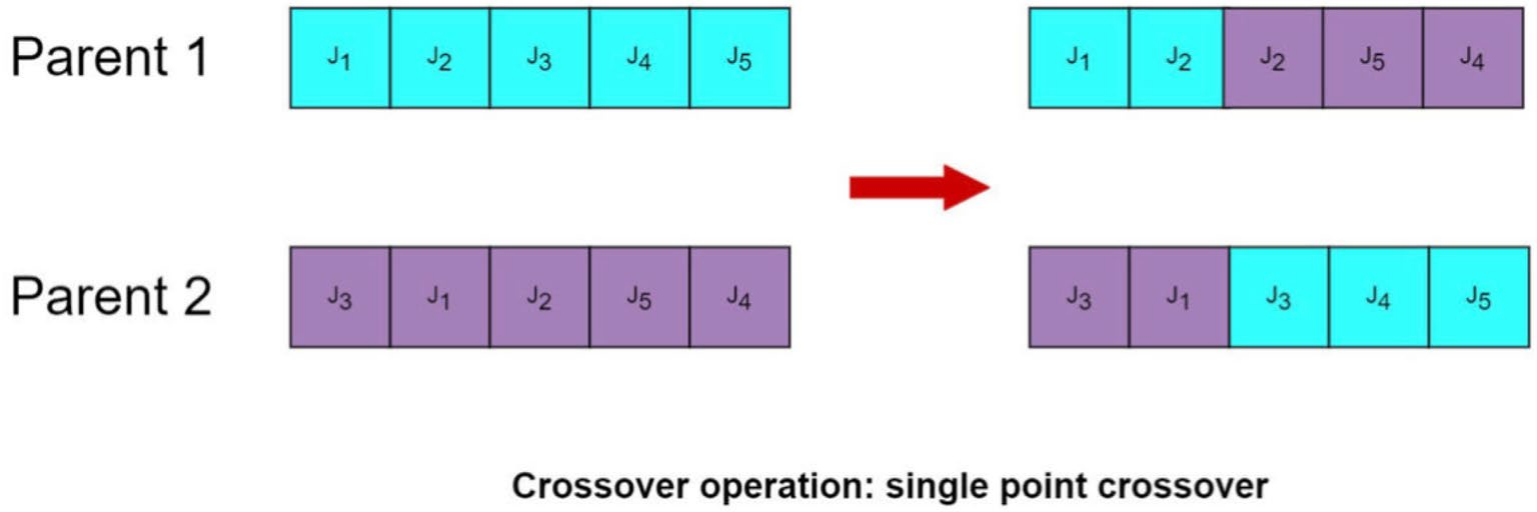
Cross operation.

In order to overcome this limitation, a best retention selection strategy from the genetic algorithm was incorporated after generating a new offspring wolf population (Fig. 7). This strategy increases the likelihood of inheriting the best individuals from the parent population to the next generation and specifically involves performing crossover operations on the top three optimal individuals.

**Figure 8:**
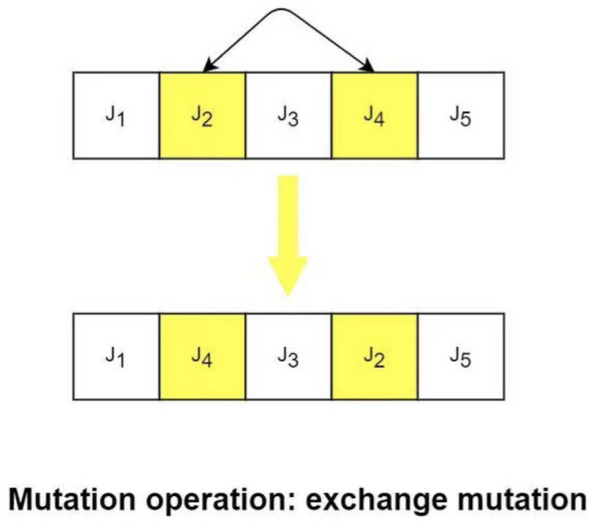
Mutation operator.

**Figure 9:**
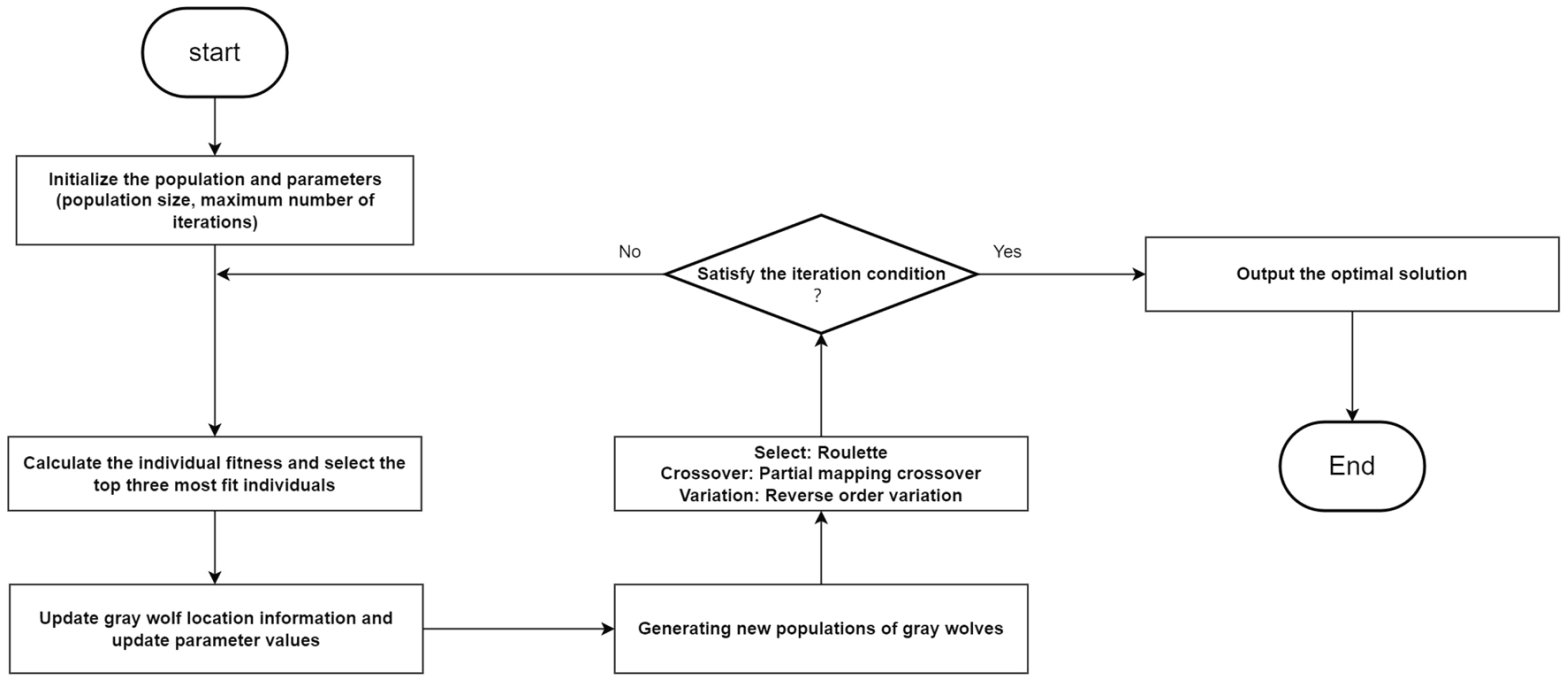
Algorithm flow chart.

To prevent the occurrence of local optima in the later iterations of the algorithm, a variation operator is introduced to perform variation operations on the elite individuals within the population (Fig. 8). This helps enhance the diversity of the population and improves the global search capability, leading to faster convergence compared to the genetic algorithm and hybrid algorithm. The flow chart of the genetic grey wolf fusion algorithm is depicted in Fig. 9.

The genetic grey wolf algorithm is able to determine the minimum time needed for all processing stages of a specific batch of prefabricated components and minimize the time difference between processing completion and delivery. This algorithm effectively organizes the production sequence of processing stages and is applicable to situations involving various types of prefabricated components and complex processes. As a result, it effectively addresses the challenges associated with scheduling the production of prefabricated components.

## Case study

### Project overview

A residential project in Hubei Province was selected as the research focus, consisting of a 21-floor building with a construction area of 50,858.8 m^2^. The first floor was constructed using cast-in-place methods, while the 2nd to 21st floors utilized prefabricated assembly structures. Data for the production scheduling optimization was obtained through field surveys of the prefabricated component production plant and interviews with the project scheduling manager. This data included information on component sizes, mold types, storage cost calculations, and the production capacity of prefabricated components on a specific working day. A subset of 12 prefabricated components from the production plan was used for the optimization analysis. The relevant data for the prefabricated component production scheduling model can be found in Table 2. The production plant operates for 8 h during a normal working day, with an additional 16 h designated as non-working time. Overtime is limited to a maximum of 4 h. Each day, 8 h are dedicated to the maintenance of the prefabricated components, during which the components produced on that day undergo maintenance in the kiln. It is important to note that the production process for prefabricated components cannot span across multiple working days.

**Table 2:** Production scheduling model data information.

Component serial number	Mold type	Processing time of each process (h)						Building information	
		N_1_	N_2_	N_3_	N_4_	N_5_	N_6_	Number of buildings	Number of layers
A	M1	0.5	0.6	0.3	0.4	10	0.2	2	1
B	M2	0.4	0.5	0.3	0.3	10	0.2	2	2
C	M1	0.5	0.3	0.4	0.4	10	0.2	2	1
D	M2	0.6	0.4	0.5	0.4	10	0.2	2	2
E	M3	0.5	0.2	0.3	0.3	10	0.2	2	1
F	M3	0.3	0.2	0.3	0.2	10	0.2	1	2
G	M1	0.6	0.4	0.5	0.4	10	0.2	1	2
H	M2	0.4	0.5	0.3	0.3	10	0.2	1	1
I	M2	0.5	0.2	0.3	0.3	10	0.2	1	1
J	M3	0.3	0.2	0.3	0.2	10	0.2	1	2
K	M1	0.5	0.3	0.4	0.4	10	0.2	1	2
L	M2	0.6	0.4	0.5	0.1	10	0.2	1	2
M	M1	0.5	0.4	0.4	0.2	10	0.2	2	1
N	M2	0.4	0.5	0.3	0.3	10	0.2	2	2
O	M3	0.5	0.4	0.4	0.2	10	0.2	2	1
P	M2	0.6	0.3	0.4	0.1	10	0.2	1	1
Q	M1	0.4	0.5	0.3	0.3	10	0.2	1	2
R	M2	0.5	0.4	0.3	0.1	10	0.2	1	1
S	M2	0.4	0.5	0.4	0.2	10	0.2	2	1
T	M1	0.3	0.4	0.4	0.3	10	0.2	2	1
U	M3	0.5	0.3	0.4	0.1	10	0.2	1	2
V	M3	0.3	0.4	0.3	0.2	10	0.2	2	2
W	M1	0.4	0.5	0.5	0.1	10	0.2	2	2
X	M2	0.5	0.3	0.4	0.3	10	0.2	1	2

Only 4 components could be produced initially due to a shortage of temporary storage resource. It took 0.7 h to wait for the remaining resources to become available before the production of the 5th component could resume. Once the resources supply were sufficient, there were no further delays. Similarly, there was a quality check after removaling the first 4 components, resulting in a waiting time of 0.3 h.

### Optimization implementation based on genetic grey wolf algorithm

#### Minimizing storage costs

The precast concrete component production scheduling model proposed in this research is implemented using MATLAB 2019. The optimization objectives are to minimize both the duration and storage cost. The parameter settings for the Genetic Algorithm are adjusted based on previous studies, resulting in the following settings: population size of 50, maximum number of iterations of 200, crossover probability of 0.6, and variation probability of 0.1.

**Figure 10:**
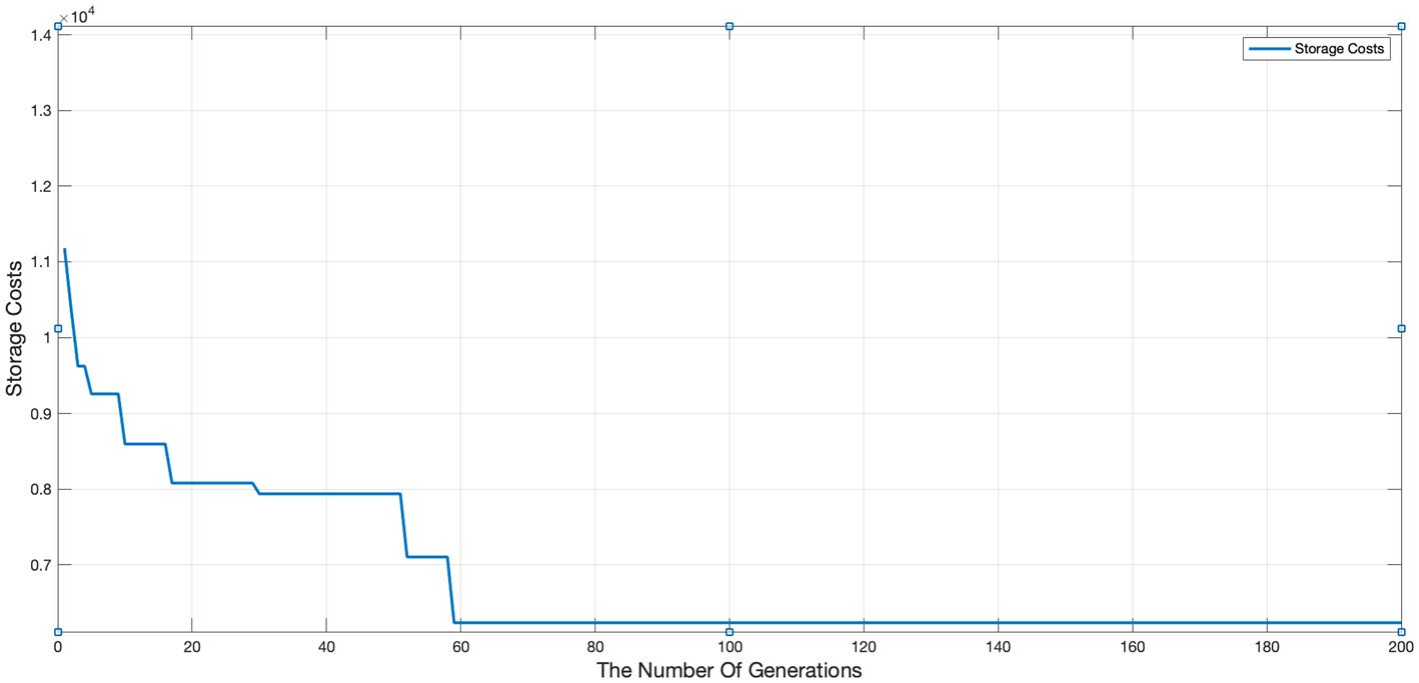
Storage cost optimization trends.

**Figure 11:**
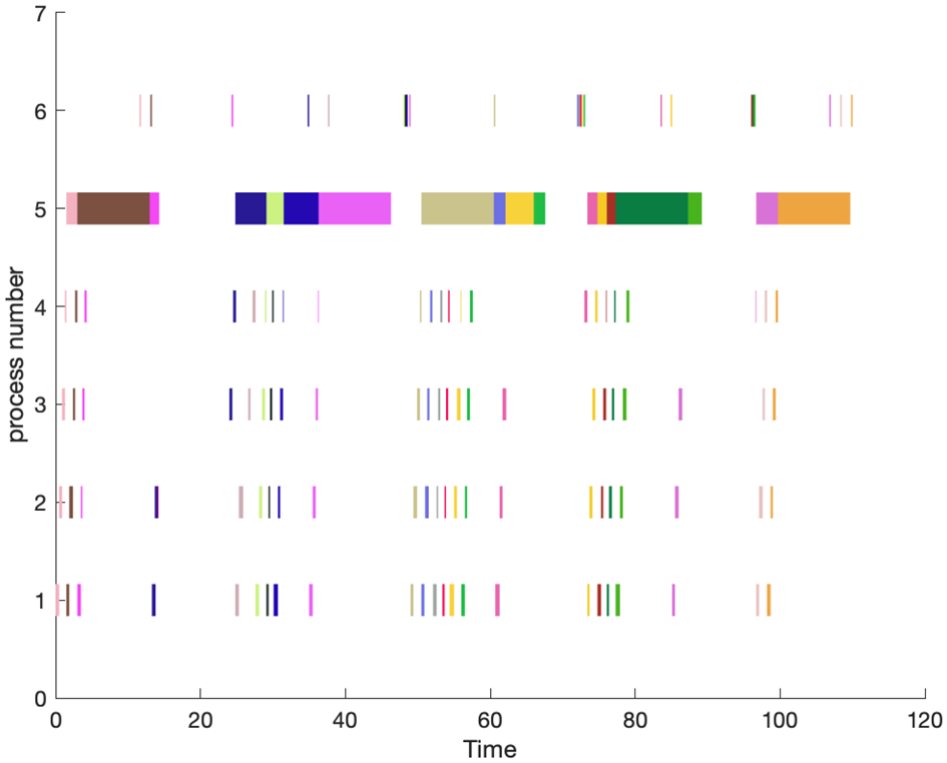
Gantt chart of minimum completion time scheduling plan.

In this section, the production scheduling is performed for the target using the same algorithm parameter settings. The MATLAB simulation provides the production scheduling information for the corresponding case. To compare the results, the manager’s experience-based component processing sequence scheme is also considered. The storage cost trend, taking resource constraints into account, is illustrated in Fig. 10. Additionally, the production information before and after optimization is presented in Table 3, and the corresponding production scheduling Gantt chart is shown in Fig. 11.

**Table 3:** Production scheduling plan information.

Number of iterations	Component processing sequence	Number of molds			Storage cost (yuan)	Completion time (D)
		M1	M2	M3		
1	TRGQVHBMUJIDPSCNFWOXEKAL	1	2	2	11,579	9
20	NGDTOSAIMHWRPJFKEULXQCVB	1	2	2	8120	7
80	POAHTECSUJRMBGNIFQKVWXLD	1	2	2	7483.5	7
160	HPCAIROTULEBSMVWFQNGKDXJ	1	2	2	6696	6
190	SIARKPTHEOGNMUVCXDFJBQLW	1	2	2	6237	5

#### Comparative analysis of genetic grey wolf algorithm and other intelligent optimization algorithms

**Figure 12:**
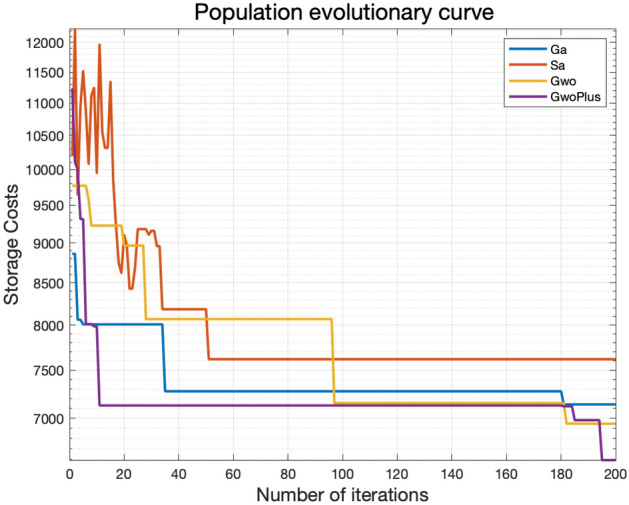
Machining curves of four scheduling methods.

To provide a clearer demonstration of the effectiveness and superiority of the GAGWO algorithm in optimizing the storage cost of component production, we compare its performance with three other algorithms: genetic algorithm, simulated annealing algorithm (SA), and grey wolf algorithm. The results of these four algorithms are compared and presented in Table 4, and Fig. 12 visually illustrates the comparison of their running outcomes.

**Table 4:** Comparison of intelligent algorithm optimization.

Optimization method	Component processing sequence	Number of molds			Storage cost (yuan)	Completion time (D)
		M1	M2	M3		
GA	MIEOCPAHLQRSTUGKVNWJDFXB	1	2	2	7312	6
SA	NODQKBFIPWHETVRAGULXJCMS	1	2	2	7623	5
GWO	PCROQDUWHISTLEJMBGFKXVNA	1	2	2	7123	5
GAGWO	SIARKPTHEOGNMUVCXDFJBQLW	1	2	2	6237	5

### Analysis of results and discussion

#### Analysis of results

Figure 10 illustrates that the adoption of the whole layer delivery mode for scheduling the production sequence of prefabricated components effectively reduces inventory costs. Analysis of Table 3 reveals that the actual production inventory cost exhibits a partial correlation with the production duration, indicating potential for optimizing inventory costs. Through iterative algorithm optimization, the storage cost is reduced from $11,579 to $6237 in the case of completion time.

The screened solutions presented in Table 4 demonstrate that both the SA algorithm and the GAGWO algorithm can significantly shorten the production period, leading to the shortest delivery time. This highlights the superior performance of the GAGWO algorithm in achieving the target of minimizing the total production period for prefabricated components. Furthermore, the genetic algorithm optimization, without considering the duration, yields the production solution with the lowest storage cost, amounting to $7312 in this case. In contrast, the GAGWO algorithm guarantees the shortest delivery time and achieves a selected prefabricated component production solution with a storage cost of $6237, approaching the minimum storage cost for this particular production scenario. Therefore, the optimization capabilities of the GAGWO algorithm enable the selection of solutions based on the shortest delivery time and the achievement of the lowest storage cost, effectively meeting the requirements of practical production.

#### Discussion

The results obtained from the case study validate the feasibility of the approach proposed in this paper. By analyzing the impact of production scheduling sequence and delivery characteristics on completion time and storage costs, the empirical production scheduling plan is optimized to achieve shorter completion time and reduced storage costs.

Based on the findings, several suggestions are proposed to enhance the management of precast plants and facilitate informed decision-making.

The objective of production scheduling is to achieve a favorable balance between on-time delivery, shortened customer lead times, and maximized resource utilization. However, the current scheduling practices in precast production are simplistic and heavily reliant on experiential knowledge, resulting in inefficient resource allocation and delayed deliveries. The model presented in this study addresses these issues.

This study is the first to consider the constraints imposed by delivery methods in precast production scheduling. Specifically, the constraint of whole floor delivery is incorporated into the scheduling process. Experimental results demonstrate that delayed delivery time and increased storage costs are observed when there is insufficient parallel production time between buildings, i.e., when the production building exceeds the duration of the in-production building. However, if there is sufficient parallel production time between buildings, the number of components produced in parallel has no significant effect. These findings underscore the critical importance of ensuring parallel production across multiple buildings in precast production scheduling.

This research introduces a hybrid genetic grey wolf algorithm to optimize the production scheduling of components on a production line. The algorithm takes into account factors such as maximum daily production efficiency, completion time, delivery time, production resources, and deployment time to improve resource utilization, reduce storage costs, and align with practical production requirements.

While this study successfully achieves its research objectives, there are still limitations to consider. Firstly, the resource production constraint examined in this study is not exhaustive. Additionally, the production scenario considered assumes an ideal condition where the production line only produces a single item and components of the same type simultaneously. Further studies are needed to supplement and enhance these aspects.

## Conclusion

This study focuses on the scheduling of precast concrete component production, considering the resource constraints and aiming to minimize the total duration and storage costs. The genetic particle swarm algorithm is employed to solve this scheduling problem. Furthermore, the study conducts a comparative analysis of optimization results obtained from different intelligent algorithms. Based on the analysis of the case results, the following conclusions are drawn:

1. The genetic grey wolf algorithm effectively identifies the optimal processing sequence for prefabricated components with multiple molds and constraints, taking into account delivery characteristics. It successfully addresses the issue of storage costs by minimizing the total production duration and the difference between production and delivery times. The proposed efficient scheduling method, exemplified by the GAGWO algorithm, demonstrates its capability to handle complex scheduling problems in precast component processing, resulting in the identification of the optimal processing sequence for prefabricated components.
2. When scheduling the production of precast concrete components, it is crucial to consider the impact on production costs resulting from the mobilization of resources such as labor, materials, and machinery, as well as the storage costs incurred during the project delivery phase. This comprehensive consideration leads to cost reduction.
3. By selecting the optimal solution for storage costs without compromising the completion time, further cost reduction and improved efficiency can be achieved.

### Supplementary Information

Supplementary Information.

## Data Availability

The datasets used during the current study available from the corresponding author on reasonable request.
