# Supplementary material for: Production scheduling of prefabricated components considering delivery methods
Source: Sci Rep. 2023 Sep 12;13:15094. doi: 10.1038/s41598-023-42374-w (PMC10497527; doi:10.1038/s41598-023-42374-w)
Supplement: Supplementary file 1 — Supplementary Information. [file 41598_2023_42374_MOESM1_ESM.zip › Src/Code Description.docx]

crossoverOperation.m crossover operation

eliteStrategy.m eliteStrategy

evolveIndividualGWO.m Evolving Individual Operations in the Gray Wolf Algorithm

evolvePopulationGWO.m Evolutionary Wolf Operations in the Gray Wolf Algorithm

GA.m Genetic Algorithms

getBestIndividualAndFitness.m Finds the best individual, optimal value in a population

getEliteIndividual.m Gray Wolf Algorithm for Finding Three Wolves in Action

getFitness.m Calculate population fitness

GWO.m Gray Wolf Algorithm

GWO_Plus.m Improved Gray Wolf Algorithm

initialPopulation.m Initializing Population

initModel2.m Initialize model, load data, etc.

learnOperation.m learnOperation

metropolisRule.m Metropolis criterion in simulated annealing algorithms

mutationOperationOfReal.m mutation operation

repairOperation.m Species Repair Operation

SA.m Simulated Annealing Algorithm

selectionOperationOfTournament.m Binary Tournament Selection Operation

showEvolCurve.m Plotting population evolution curves

showGantt.m Simplified Gantt Chart

showIndividual.m Plotting Individuals (out of map)

updateIndividualStrategyGWO.m Suboperations for Evolving Individuals in the Gray Wolf Algorithm
